# Influence of Infographics on Consumer Perception of Organ-Enhanced Ground Beef

**DOI:** 10.3390/foods15173012

**Published:** 2026-08-27

**Authors:** Savannah L. Douglas, Autumn L. Armaly, Rilee A. Bennett, Don R. Mulvaney, Jase J. Ball, Soren P. Rodning, Jason T. Sawyer

**Affiliations:** Department of Animal Sciences, Auburn University, Auburn, AL 36849, USA; sld0060@auburn.edu (S.L.D.); ala0084@auburn.edu (A.L.A.); rzb0145@auburn.edu (R.A.B.); mulvadr@auburn.edu (D.R.M.); jjb0127@auburn.edu (J.J.B.); rodnisp@auburn.edu (S.P.R.)

**Keywords:** consumer perception, educational infographic, ground beef, organ meats, value-added

## Abstract

Consumer acceptance remains a primary barrier to incorporating organ meats into value-added beef products, despite their nutritional profile. This study evaluated the effectiveness of educational infographics emphasizing either nutritional benefits or economic value on consumer perceptions of organ-enhanced ground beef. A total of 1939 U.S. consumers served as the experimental unit and completed an online survey administered through Qualtrics. Participants were randomly assigned to one of two educational infographic treatments (nutrition or economics and resource utilization). Consumer perception indices were calculated before and after infographic exposure. Changes in perception (Δ) were analyzed with infographic treatment, food familiarity level and their interaction. Educational infographics produced small numerical improvements in consumer perception, although responses did not differ between infographic types. No differences were observed among nutrition- or economic-focused infographics for any perception index (*p* = 0.1570). Likewise, no interaction between infographic type and Food Familiarity Index were detected (*p* = 0.4849). However, food familiarity significantly influenced acceptance (*p* < 0.0001), label transparency (*p* < 0.0001), and willingness-to-pay (*p* < 0.0001). Consumers with greater food familiarity reported more favorable perceptions than those with lower familiarity. However, FFI level did not impact economic perception. Current results demonstrate that concise educational messaging can influence consumer perceptions of organ-enhanced ground beef. Furthermore, improving consumer food familiarity may enhance acceptance and marketability of nutrient-dense beef products containing edible organ meats.

## 1. Introduction

Ground beef remains one of the most widely consumed beef products in the United States due to its affordability, versatility and widespread availability [1,2]. As consumers seek foods providing both nutritional benefits and economic value, there has been a heightened interest in developing nutritionally dense food products without substantially increasing consumer cost [3,4]. Simultaneously, the livestock industry continues to face pressure to enhance sustainability by maximizing utilization of the entire carcass and reduce food waste [5,6]. Identifying practical methods for altering the value of edible beef by-products while maintaining consumer acceptance has become an important area of research [4,6].

Maximizing utilization of edible organs aligns with several sustainability goals promoted throughout the beef industry. Whole-carcass utilization improves resource efficiency by generating greater value from food animals entering the supply chain while reducing the amount of edible products entering export markets, pet food production, or rendering [5]. Furthermore, as the U.S. cattle inventory is currently at its lowest in decades, maximizing value from existing carcasses has become increasingly important for maintaining beef supply while improving production efficiency [7]. Rather than increasing production, incorporation of nutrient-dense edible by-products into commonly consumed products offers an opportunity to improve nutritional quality while maximizing existing resources. This approach supports growing consumer and industry interest in sustainable food systems that balance environmental stewardship with affordability and increased nutritional quality [6,8].

Research on educational messaging can influence consumer perceptions of novel foods by improving understanding of nutritional benefits and production practices [9,10]. Infographic-based educational materials provide concise visual communication that allows consumers to process information more efficiently than traditional text-based educational approaches [11]. When consumers receive information regarding food production, sustainability, or health attributes, their attitudes and purchasing intentions tend to improve [10]. However, the magnitude of these effects vary depending on the message presented and consumer’s existing beliefs [12,13]. For organ-enhanced meat products, educational materials emphasizing nutritional advantages or economic value may reduce negative perceptions associated with organ consumption by reframing these ingredients as functional, nutrient-rich components rather than undesirable by-products. However, whether nutritional information or economic value serves as a more effective educational strategy for improving perceptions of organ-enhanced meat products remains largely unknown.

Previous studies have evaluated consumer acceptance of organ meats and other protein sources such as fish, veal, and pork [12,14]. Research has not investigated how educational messages could influence consumer perceptions of organ-enhanced ground beef. Understanding which educational approaches most effectively communicate the benefits of organ inclusion may assist industry stakeholders in developing marketing strategies that improve acceptance while supporting sustainable meat production.

Beyond nutritional enhancement, incorporation of edible organ meats into familiar meat products represents an opportunity to address several emerging challenges facing modern food systems. Increasing demand for sustainable protein production, pressure to improve whole-carcass utilization, and growing interest in nutrient-dense and affordable foods have intensified efforts to identify value-added uses for edible by-products [5]. Successful implementation depends not only on product formulation but also on consumer willingness to purchase and consume these products.

Therefore, the objective of this study was to evaluate the effects of two informational infographics—nutrition and economics and resource utilization—on consumer perceptions, willingness-to-pay, labeling preferences, and perceived nutritional value of organ-enhanced ground beef. Specifically, the infographics emphasized either nutritional attributes or economic and resource utilization attributes. Additionally, this study examined whether consumers with differing levels of food familiarity respond differently to each educational topic.

## 2. Materials and Methods

### 2.1. Survey Development and Participant Recruitment

This study was approved by the Auburn University Institutional Review Board (STUDY00001161). An online survey was developed using Qualtrics (Qualtrics, Provo, UT, USA) to evaluate consumer perceptions of ground beef products containing organ meats and the influence infographic-based educational messaging on those perceptions. Treatments consisted of two infographics: (1) nutritional benefits and (2) economic benefit of organ meats. The survey consisted of demographic questions, Likert-type scales, multiple choice questions, and willingness-to-pay assessments. A total of 1939 participants completed the survey. All participants provided informed consent prior to completion of the survey.

Participants were recruited through Prolific (Prolific Academic Ltd., London, UK). This study aimed for a representative sample of the United States population. Eligibility requirements for the survey included being at least 18 years of age, consuming ground beef two to three times per month, and participating in grocery purchasing decisions within their household (Table 1). Survey questions consisted of demographic questions, Food Familiarity Index (FFI) items, consumer perception statements, willingness-to-pay, labeling preference, and infographic evaluation questions. Consumer perception questions were given both before and after infographic exposure to quantify changes in participant response compared to the educational intervention.

### 2.2. Survey Design

This survey consisted of three phases: a baseline assessment, exposure to educational graphics, and a post-exposure assessment. Upon entering the survey, participants were randomly assigned by the Qualtrics randomization function to view either the nutrition-focused or economic-focused infographic. Random assignment occurred automatically and each participant viewed only one infographic before completing the post-exposure assessment. Viewing time and exposure conditions were controlled within Qualtrics by requiring participants to view the assigned infographic for a minimum of 15 s before they were permitted to advance to the subsequent questions. After viewing the assigned infographic, participants’ comprehension was assessed using a clarification question. Participants who answered this question incorrectly were excluded from the data set. During the baseline assessment, participants completed demographic questions and the Food Familiarity Index, followed by questions evaluating five constructs: acceptance, nutrition perception, economic perception, labeling preferences, and willingness-to-pay. Baseline assessment questions included statements such as “Ground beef is a nutritious food,” “Ground beef is a good value for the price,” “Organ meats (liver, heart, or kidney) are nutritious foods,” “The idea of eating organ meats is unpleasant to me,” and “I would need clear labeling that indicates the product contains organ meats before purchasing it.” Participants were also asked about their willingness to consume and purchase organ-enhanced ground beef, willingness-to-pay, required price discounts, and whether they would consider purchasing the product if it were offered at a lower price than regular ground beef. Negatively worded items, including unpleasantness, were reverse-coded prior to calculation of the composite acceptance index so that greater scores consistently represented greater consumer acceptance. The Food Familiarity Index was adapted from previously published studies [15,16]. Survey items were developed based on the previous literature evaluating consumer acceptance of novel meat products, food labeling, nutrition communication, and willingness-to-pay [17,18], while questions regarding prior organ meat consumption and typical ground beef purchasing habits were developed specifically for this study. Participants rated their agreement with statements using a 7-point Likert scale; (1 represented strong disagreement and 7 represented strong agreement). Additional questions assessed willingness-to-pay, required price discounts, and information needs related to product labeling and nutrition. Willingness-to-pay was measured using a six-point scale ranging from 1 (I would not try the product at any price) to 6 (I would pay up to 20% more), with intermediate response options of no additional cost, up to 5% more, up to 10% more, and up to 15% more. Required price discounts were measured using a six-point scale ranging from 1 (I would not try the product at any price) to 6 (no discount needed), with intermediate options of a 5%, 10%, 15%, or 20% (or greater) price reduction. Information needs related to product labeling and nutrition were assessed using a 7-point Likert scale (1 = strongly disagree; 7 = strongly agree).

**Figure 1:**
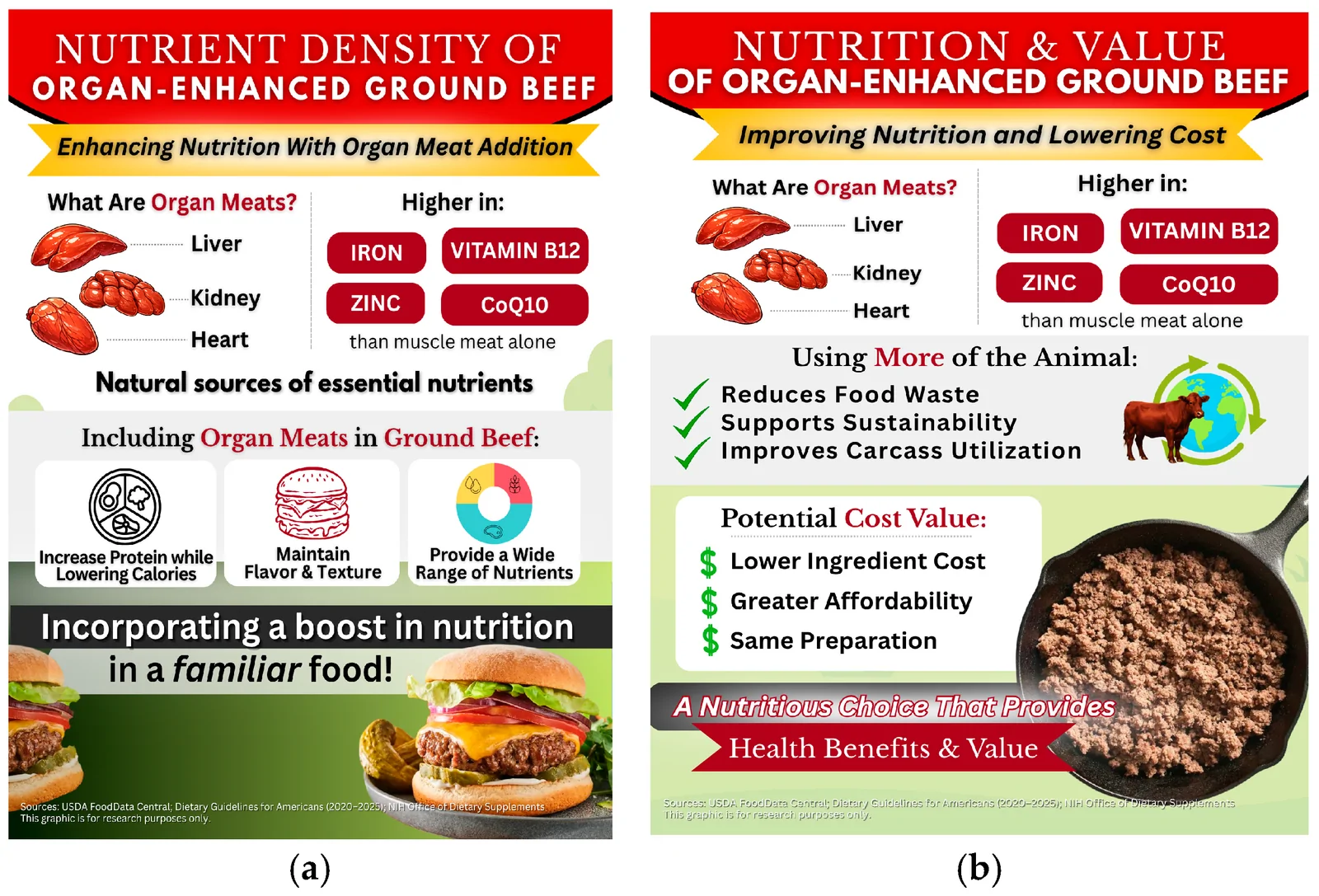
(**a**) Nutritional infographic of organ-enhanced ground beef viewed by survey participants. (**b**) Cost and value infographic of organ-enhanced ground beef viewed by survey participants.

The nutrition infographic highlighted the nutritional attributes of organ meats, including protein, vitamins, and minerals (Figure 1a). An infographic highlighting economics and resource utilization focused on economic considerations associated with organ meat inclusion, including affordability and product value (Figure 1b). Participants viewed their assigned infographic and completed a post-treatment survey consisting of the same organ meat perception questions given during the baseline assessment. Following survey questions, participants completed a heat map activity embedded within the survey in which they selected the portions of the graphic they found most impactful or informative. Responses were used to generate visual heat maps illustrating areas of greatest participant interest.

### 2.3. Response Indices

Consumer perception outcomes included three composite indices (acceptance, economic perception, and need for labeling information) and two single-item measures (nutrition perception and willingness-to-pay). Composite indices were constructed by grouping items intended to assess related aspects of each consumer perception area. Responses were used to calculate composite perception indices. An acceptance index was calculated as the mean of willingness-to-consume, purchase-likelihood, and unpleasantness scores. An economic perception index was calculated using responses related to economic value and price-based acceptance. A labeling perception index was calculated from questions pertaining to the importance of ingredient disclosure and nutrition information. Willingness-to-pay and nutrition perception were analyzed separately from the composite indices. Willingness-to-pay responses were coded such that greater values represent greater willingness to pay a premium for products containing organ meats.

For each perception variable, pre-treatment and post-treatment scores were calculated as means. Delta values were calculated as delta = post-treatment score − pre-treatment score, where positive values indicate improved perceptions following infographic exposure.

### 2.4. Food Familiarity Index

The Food Familiarity Index (FFI) consisted of seven questions assessing participants’ familiarity with food production, nutrition, agricultural marketing, and organ meats (Table 2). Index was adapted from previous food familiarity and agricultural literacy research [15,16]. Responses were scored on a 10-point scale (1 = least familiar; 10 = most familiar) and summed to produce an overall FFI score ranging from 7 to 70. Internal consistency of the FFI was assessed using Cronbach’s alpha, with the seven-item index demonstrating good reliability (α = 0.87) [19].

### 2.5. Statistical Analysis

Survey data were collected from Qualtrics and analyzed using PROC GLIMMIX in SAS version 9.4 (SAS Institute Inc., Cary, NC, USA). This study was a completely randomized design with two infographic treatments: economics and resource utilization-focused and nutrition-focused. The experimental unit was the individual survey respondent, as each participant was randomly assigned to one infographic treatment and provided one independent set of responses. Perception indices were calculated before and after infographic exposure, and changes in perception (Δ = post-treatment score − pre-treatment score) were used as response variables to evaluate the change in perception following infographic exposure. Infographic treatment, Food Familiarity Index (FFI) category (low, medium, or high), and their interaction were included as fixed effects. Since established cut points for this Food Familiarity Index have not been validated, FFI scores were categorized into thirds based on the possible score range: low familiarity = 7 to 28, medium familiarity = 29 to 49, and high familiarity = 50 to 70. When significance was observed (*p* < 0.05), least-square means were generated using the LSMEANS statement and separated using pairwise comparisons.

## 3. Results and Discussion

### 3.1. Food Familiarity Index

Participants were classified into low, medium and high Food Familiarity Index (FFI) categories based on their response to seven questions assessing familiarity with food production, nutrition, marketing and organ meats (Table 3). Nearly equal distribution of respondents among the three categories indicates that the survey captured consumers with a broad range of food-related knowledge and experiences. Furthermore, the seven-question FFI demonstrated internal consistency (Cronbach’s a = 0.87), indicating the questions measured a common construct related to food familiarity [20].

Although most participants were classified as having moderate food familiarity, respondents represented a broad spectrum of familiarity levels, allowing for evaluation of whether educational messaging was perceived differently across consumers with varying degrees of food-related knowledge. Food familiarity has been recognized as an important factor influencing consumer perceptions because previous knowledge and experiences affect how individuals interpret information regarding food production, nutrition, and novel food products [14,20]. Consumers with greater familiarity are often better equipped to evaluate product attributes, whereas individuals with lower familiarity may rely more heavily on preconceived beliefs or food neophobia when encountering unconventional ingredients [12,14]. FFI score frequencies represent a broad spectrum of food familiarity. This allowed for subsequent analyses to evaluate whether educational messaging influenced consumer perceptions similarly across differing levels of prior food knowledge. This approach provides additional insight into whether educational interventions may be equally effective across diverse consumer populations or whether familiarity with food systems contributes to differences in consumer acceptance of organ-enhanced ground beef.

From a science communication perspective, educational infographics represent an initial step in the transfer of knowledge rather than a direct mechanism for changing consumer behavior. Educational interventions may first increase awareness and understanding of product attributes before influencing attitudes, purchase intentions, or food choice behaviors [10,11]. Consequently, evaluating food familiarity alongside educational messaging provides insight into whether consumers simply recognize new information or whether that information is sufficient to influence perceptions of novel meat products. Distinguishing these stages of consumer processing is important, as improved knowledge does not necessarily translate into immediate behavioral change, particularly for unfamiliar foods associated with perceived risk or uncertainty [12,17].

### 3.2. Influence of Food Familiarity

No significant interactions were observed for infographic treatment × food familiarity scores for acceptance (*p* = 0.4168), economic perception (*p* = 0.1000), nutrition perception (*p* = 0.7695), labeling transparency (*p* = 0.4262), or willingness-to-pay (*p* = 0.3249). This indicates that consumers respond similarly to the educational infographics regardless of food familiarity level. Although familiarity influenced overall perceptions of organ-enhanced ground beef, it did not alter how participants responded to the educational messages themselves. However, significant main effects of the Food Familiarity Index were observed (Table 4) for acceptance (*p* < 0.0001), nutrition perception (*p* < 0.0001), label transparency (*p* < 0.0001), and willingness-to-pay (*p* < 0.0001), whereas economic perception (*p* = 0.8270) was not influenced by FFI.

Acceptance for organ meat consumption increased with a greater level of food familiarity. Acceptance scores (*p* < 0.0001) increased from 3.24 in the low-familiarity group to 3.95 and 4.63 for the medium- and high-familiarity groups. This suggests that consumers possessing greater familiarity with food production and agricultural systems may be associated with greater acceptance or receptiveness toward unconventional food products. It is plausible that prior knowledge reduces uncertainty surrounding production practices, ingredient functionality, and nutritional value. Consequently, these individuals may rely more heavily on objective product attributes than emotional reactions when evaluating unconventional foods. In contrast, consumers with lower food familiarity may experience greater food neophobia or perceive greater uncertainty, making acceptance more resistant to change despite receiving identical educational information [12,21]. Although not directly measured within the current study, similar relationships between food familiarity, agricultural literacy, and consumer acceptance have been reported previously, where greater knowledge of food production was associated with increased willingness to purchase and consume novel food products [14,16]. These findings suggest that educational messaging may improve consumer perception, whereas broader acceptance may require increasing familiarity through repeated educational exposure and direct product experiences.

Participants reported similar perceptions of the economic value of organ-enhanced ground beef regardless of their level of food familiarity (*p* = 0.8270). These findings suggest that educational background or familiarity with food systems alone may not influence consumers’ perceptions of the economic benefits associated with utilizing edible organ meats. Economic considerations may be more dependent on product price, perceived quality and personal purchasing priorities. In contrast, the literature suggests that consumers with greater knowledge of food systems may better recognize the potential economic value of utilizing edible organ meats as nutrient-dense ingredients capable of increasing carcass utilization while reducing waste [6,8,16]. Although food familiarity did not influence economic perception (*p* = 0.8270), this finding highlights that economic evaluations may be driven by factors beyond food knowledge alone. Consumers frequently evaluate food purchases using perceived product quality, household budgets, expected eating experiences, and price sensitivity, in addition to objective information [13]. Consequently, greater familiarity with food production may not necessarily translate into greater perceived economic value of organ-enhanced products.

Label transparency improved with heightened food familiarity (*p* < 0.0001). Participants with greater food familiarity reported greater need for nutritional information and product labeling than consumers with low familiarity. Survey respondents who possessed greater knowledge of food production may have increased desire for information when interpreting ingredient statements and nutritional claims. Moreover, greater food familiarity may reduce skepticism toward products containing unfamiliar ingredients. In contrast, consumers with limited food knowledge often rely on simple heuristics or on unfamiliar ingredient names when making purchasing decisions, which may reduce confidence in product labeling [11,17].

Nutrition perception increased with food familiarity (*p* < 0.0001). Participants with greater food familiarity may have been better equipped to interpret the nutritional information presented because prior knowledge provides context for understanding health claims and nutrient functions [10]. This suggests that familiarity enhances consumers’ ability to recognize nutritional value but does not necessarily imply that nutrition knowledge alone is sufficient to alter purchasing behavior.

Consumers with high food familiarity consistently reported greater willingness-to-pay than those with medium or low familiarity (*p* < 0.0001). Although educational messaging may improve perceived nutritional benefits, willingness-to-pay represents a more complex stage of consumer decision-making than product recognition alone. Purchasing decisions are influenced by expected taste, familiarity, trust, perceived risk, social norms, and personal values, in addition to nutritional information [12,14]. Consequently, a single educational exposure may be sufficient to increase perception but insufficient to overcome established purchasing habits or concerns associated with unfamiliar ingredients. Repeated educational campaigns, combined with opportunities for direct product experience or tasting demonstrations, may be more effective in improving long-term consumer acceptance and willingness-to-pay.

Collectively, these findings demonstrate that food familiarity influences how consumers perceive organ-enhanced ground beef, whereas educational messaging was interpreted similarly across familiarity groups. From a science communication perspective, these results suggest that concise educational materials can effectively increase perception among diverse consumer audiences; however, perception of a product should not be interpreted as synonymous with consumer acceptance or purchasing behavior. Future educational initiatives may benefit from combining repeated evidence-based messaging with direct product experiences, extension programming, retail demonstrations, and digital communication strategies to increase familiarity and reduce uncertainty surrounding organ-enhanced meat products.

### 3.3. Consumer Perception

Consumer perceptions before and after exposure to the educational infographics are presented in Table 5. Prior to infographic exposure, no differences were observed between participants for acceptance (*p* = 0.1150), economic perception (*p* = 0.0760), willingness-to-pay (*p* = 0.3440), label transparency (*p* = 0.1443), or nutrition perception (*p* = 0.3142), suggesting that baseline consumer perceptions were similar between treatment groups. Establishing similar baseline perceptions is essential when evaluating educational interventions because pre-existing beliefs can strongly influence how consumers interpret food-related information and subsequently evaluate novel food products [12,14]. Changes in perception following infographic exposure (Δ) did not differ for acceptance (*p* = 0.2259), economic perception (*p* = 0.3494), willingness-to-pay (*p* = 0.9094), label transparency (*p* = 0.1570), or nutrition perception (*p* = 0.2266).

Although participants assigned to the nutrition infographic reported higher post-exposure nutrition perception scores than those assigned to the economic infographic (*p* = 0.0071), infographic type did not influence changes in nutrition perception (*p* = 0.2266). It has been reported that nutritional education has consistently been shown to improve consumers’ ability to recognize the health attributes of foods when information is presented in a concise and visually accessible format [9]. Likewise, it has been reported that simplified nutrition information improves consumer understanding and confidence during food purchasing decisions [18]. Current findings suggest that brief educational messaging may improve consumer perceptions of the nutritional value of organ-enhanced ground beef, regardless of whether the infographic emphasizes nutritional or economic benefits. Consumer acceptance of novel foods is rarely determined by nutritional information alone and instead reflects a combination of familiarity, perceived risk, expected sensory quality, trust, food neophobia and established purchasing habits [12,22]. Furthermore, it is suggested that consumers evaluating unconventional foods frequently rely on existing beliefs and prior experiences, making attitudes resistant to change following a single educational exposure [14]. Consequently, participants appeared to recognize the nutritional advantages presented in the infographics; however, increased exposure alone was insufficient to substantially alter purchasing intentions (*p* = 0.9094) or overall product acceptance (*p* = 0.2259). These findings suggest that exposure to a topic represents an early stage of consumer decision-making that may not immediately translate into behavioral change.

Both educational approaches resulted in small numerical improvements across all measured variables following exposure. Similar changes have been reported following educational interventions designed to improve consumer knowledge of food products, where increased awareness does not necessarily translate into immediate behavioral change [23]. Since organ-enhanced ground beef represents an unfamiliar product for many consumers, repeated educational exposure or direct tasting experiences may be necessary before measurable improvements in acceptance and willingness-to-pay are observed [12].

These findings are further supported by the participant-generated heat maps, which identified the nutritional claims as the most impactful components of both educational graphics. The data suggest that consumers recognized the nutritional advantages of organ-enhanced ground beef. However, increased perception was insufficient to overcome existing attitudes toward products containing organs. Similar relationships between improved nutrition knowledge and limited behavioral change have been reported previously, emphasizing that educational messaging is most effective when combined with opportunities to increase familiarity and reduce uncertainty surrounding novel foods [9,12,21].

### 3.4. Heat Map Analysis

**Figure 2:**
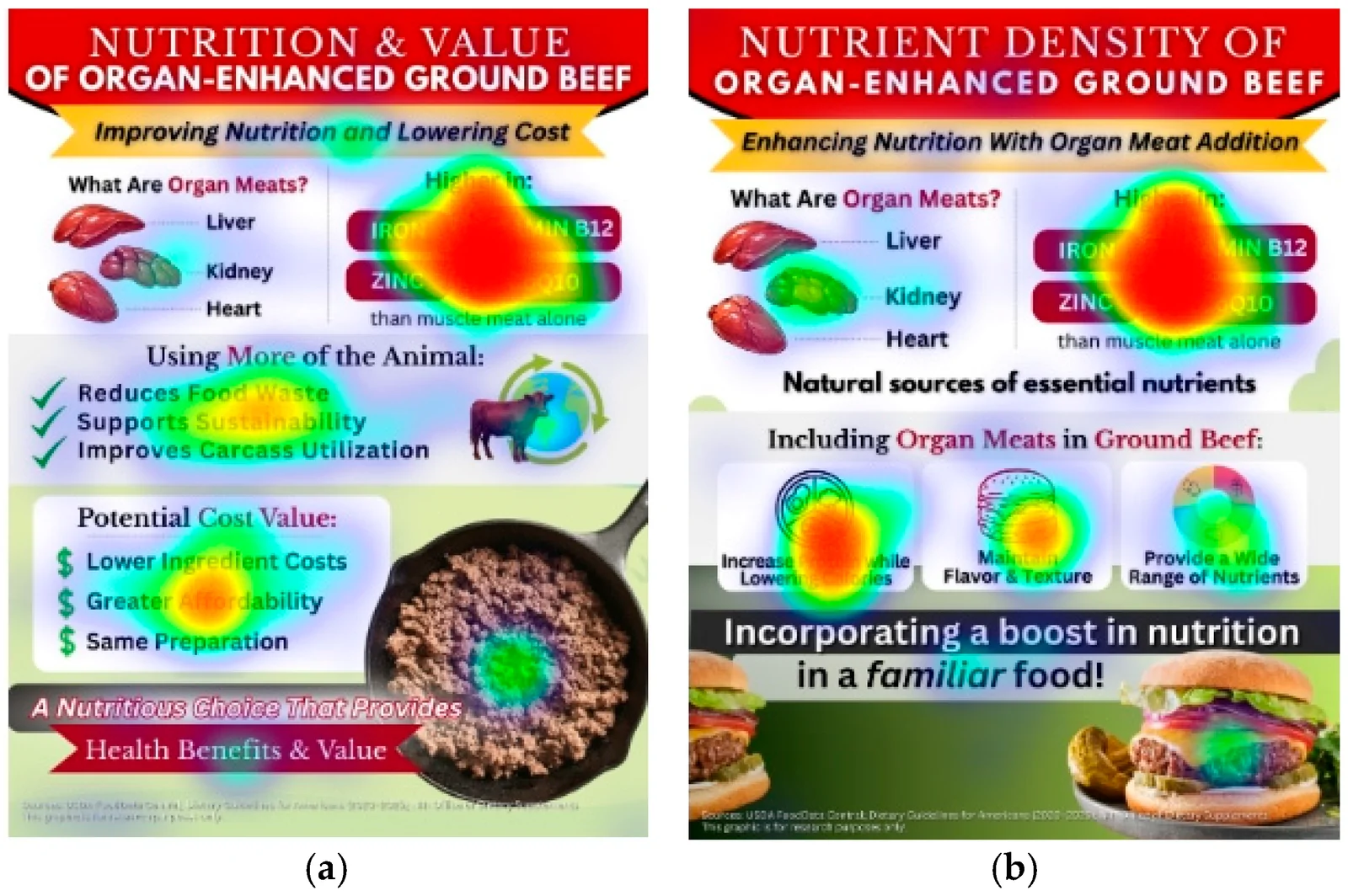
(**a**) Participant-generated heat maps of the most impactful part of the infographic treatment for economic and utilization. (**b**) Participant-generated heat maps of the most impactful part of the infographic treatment for nutrition. Warmer colors (darker red) indicate greater number of selections.

Heat map responses were used to identify which portion of each infographic participants perceived as the most impactful (Figure 2a,b). Warmer colors (i.e., darker red) indicate areas that received a greater number of participant selections. Across both infographic types, respondents consistently identified the nutritional claims highlighting the high concentration of iron, zinc, vitamin B_12_ and CoQ10 as the most influential components. This suggests that nutritional claims and visual comparisons were more memorable and persuasive than explanatory text, which is consistent with previous research demonstrating that consumers preferentially respond to simplified visual nutrition messages when evaluating food products [9,11].

Emphasis on nutritional benefits across both infographics indicates that survey respondents viewed these specific nutritional benefits as meaningful when evaluating organ-enhanced ground beef. Heat map results complement the survey results, as participants who viewed the nutritional infographic reported significantly greater post-treatment nutrition perception scores than those exposed to the graphic highlighting economic and sustainability benefits (*p* = 0.0071). However, because changes from pre- to post-treatment did not differ between infographic treatments, these differences likely reflect baseline variation rather than an effect of infographic type. Collectively, survey responses and heat maps suggest that nutritional messaging emphasizing specific functional nutrients resonated more strongly with consumers than broader messages related to affordability or sustainability. Previous research has similarly demonstrated that health-related attributes often exert a greater influence on consumer acceptance of novel food products than economic incentives alone [12,14].

Results provide practical implications for future educational materials promoting organ-enhanced meat products. Respondents consistently identified specific nutrient claims as the most impactful information; future consumer education efforts should prioritize concise, evidence-based nutritional messaging and visually emphasize key micronutrients rather than relying on lengthy explanatory text. Presenting nutrient-specific benefits in a clear graphical format may improve consumer understanding while increasing acceptance of value-added meat products containing edible organs.

### 3.5. Study Limitations

A few limitations should be considered in the findings of the current study. First, a no-exposure control group was not included because the primary objective was to compare changes in consumer perceptions between the two infographic conditions. Therefore, changes in perceptions cannot be attributed solely to infographic exposure. Additionally, differences in visual design, including layout, imagery, and color, were not independently controlled, so their influence cannot be separated from the informational content presented. Moreover, the economics and resource-utilization infographic incorporated multiple messages related to economic considerations, sustainability, and resource utilization. Therefore, the individual effects of these messages could not be determined. Finally, outcomes were measured immediately following infographic exposure and primarily reflected self-reported consumer perceptions and purchase-related attitudes. Although comprehension of the assigned infographic was assessed, long-term information retention, changes in consumer knowledge, and actual purchasing behavior were not evaluated. Future research should address these limitations by incorporating appropriate control conditions, isolating specific informational messages, evaluating longer-term responses, and determining whether changes in consumer perceptions translate to purchasing behavior.

## 4. Conclusions

Exposure to nutrition and economics and resource utilization-based infographics improved consumer perception of organ-enhanced ground beef. However, infographic type did not significantly influence changes in acceptance, economic perception, willingness-to-pay, label transparency or overall nutrition perception. Participants who viewed the nutrition infographic reported greater nutrition perception scores than those receiving the economic and resource infographic, suggesting that emphasizing specific nutrients naturally present in organ meats may increase recognition of their nutritional value. Nevertheless, increased perceived nutritional value alone was insufficient to substantially alter purchasing intentions or overall product acceptance following a single educational exposure.

Food familiarity emerged as an important factor influencing consumer responses to organ-enhanced ground beef. Consumers with greater familiarity with food production, nutrition, and organ meats consistently demonstrated higher acceptance, greater perceived nutritional value, increased need for labeling information, and greater willingness-to-pay than consumers with lower familiarity. In contrast, economic value was unaffected by food familiarity, indicating that familiarity with food and food production was not associated with consumers’ perceptions of the economic value of organ-enhanced ground beef.

Collectively, the results of this study suggest that successful commercialization of organ-enhanced ground beef will likely require integrated communication strategies that extend beyond educational messaging alone. Combining concise nutrition communication with transparent labeling, repeated consumer exposure, and opportunities for direct product experience may more effectively improve familiarity, reduce uncertainty, and increase long-term consumer acceptance.

These findings indicate that educational infographics may serve as an effective tool for increasing consumer perception of novel food products. However, educational materials alone are unlikely to alter purchasing behavior, suggesting that broader marketing strategies may be needed to promote consumer acceptance. It is important to note that the current study evaluated self-reported perceptions through an online survey rather than observed purchasing behavior. Future research should evaluate the influence of repeated educational exposure, alternative communication formats, and the impact of educational messaging on real-world purchasing decisions. Overall, results contribute to the growing field of science communication by demonstrating that educational messaging represents an important first step toward consumer exposure, but sustained changes in purchasing behavior will likely require broader consumer engagement strategies.

## Figures and Tables

**Table 1:** Demographic characteristics of participants.

	Percentage
Sex	
Male	49.59%
Female	50.34%
Prefer not to say	0.31%
Age	
18–20 years old	4.28%
21–29 years old	16.40%
30–39 years old	19.24%
40–49 years old	15.21%
50–59 years old	21.09%
60–69 years old	18.62%
70–79 years old	5.16%
80–89 years old	0.41%
Residential Area	
Rural	22.23%
Suburban	53.64%
Urban	24.76%
Grocery store most frequented	
Costco, Sam’s Club	11.09%
Kroger, Publix, or similar stores	44.25%
Local grocery store	25.79%
Online grocery (Instacart/Amazon)	5.57%
Whole Foods, Sprouts, Trader Joe’s, or similar stores	13.67%
Prefer not to say	0.31%
U.S. Region	
Midwest	21.14%
Northeast	17.12%
South	35.12%
West	23.00%

**Table 2:** Food Familiarity Index (FFI) questions presented to the participants.

FFI Item
1. I would say I know something about how most of the food I eat is raised or produced.
2. I would devote time and energy to learning about different food systems and current agricultural practices used in food production.
3. I make buying decisions based on how and/or where a specific food item was produced.
4. I seek out information about where my food comes from.
5. I make buying decisions based on the nutritional composition and health implications.
6. I am familiar with the safety, quality, and marketing factors of food.
7. I am familiar with organ meats (liver, heart, kidney) as food.

**Table 3:** Frequencies and percentages of Food Familiarity Index placement.

FFI Level	%	FFI Score ^a^
Low	21.94	21.56
Medium	54.59	38.99
High	23.48	56.57

^a^ FFI score: low = 7 to 28; medium = 29 to 49; high = 50 to 70.

**Table 4:** Main effect of Food Familiarity Index level on post-exposure consumer perception indices.

	FFI Level				
Perception Index ^a^	Low	Medium	High	SEM *	*p*-Value
Acceptance	3.24 ^c^	3.95 ^b^	4.63 ^a^	0.056	<0.0001
Economic	3.68	3.69	3.63	0.070	0.8270
Nutrition Perception	5.82 ^c^	5.94 ^b^	6.12 ^a^	0.041	<0.0001
Label Transparency	5.60 ^c^	5.80 ^b^	5.96 ^a^	0.052	<0.0001
Willingness-to-pay	1.81 ^c^	2.09 ^b^	2.47 ^a^	0.040	<0.0001

FFI Level: Low score = (7 to 33), medium scores = (34 to 45), high scores = (46 to 70). ^b, c^ Means within a row lacking a common superscript differ (*p* < 0.05). * Standard error of the mean. ^a^ Perception indices were calculated as the mean of multiple Likert-scale items, with higher values indicating more favorable consumer perceptions.

**Table 5:** Consumer perceptions pre- and post-infographic exposure.

	Infographic Type			
	Nutrition	Economic	SEM *	*p*-Value
Acceptance				
Pre	3.80	3.67	0.059	0.1150
Post	3.97	3.95	0.061	0.8066
Δ	0.17	0.27	0.063	0.2259
Economic				
Pre	3.54	3.41	0.052	0.0760
Post	3.68	3.66	0.054	0.7352
Δ	0.15	0.25	0.076	0.3494
Willingness-to-Pay				
Pre	2.10	2.06	0.030	0.3440
Post	2.13	2.10	0.032	0.5556
Δ	0.04	0.05	0.035	0.9094
Label Transparency				
Pre	5.71	5.78	0.038	0.1443
Post	5.79	5.80	0.039	0.8954
Δ	0.09	0.01	0.042	0.1570
Nutrition				
Pre	5.46	5.40	0.040	0.3142
Post	6.02 ^a^	5.89 ^b^	0.031	0.0071
Δ	0.56	0.49	0.042	0.2266

^a, b^ Means within a row lacking a common superscript differ (*p* < 0.05). * Standard error of the mean. Δ: change in perception (post survey score − pre survey score). Perception indices were calculated as the mean of multiple Likert-scale items, with higher values indicating more favorable consumer perceptions.

## Data Availability

The original contributions presented in this study are included in this article; further inquiries can be directed to the corresponding author.

## Abbreviations

The following abbreviations are used in this manuscript:

IRB	Institutional Review Board
FFI	Food Familiarity Index
SEM	Standard error of the mean
